# Supplementary material for: The economic value of augmentative exoskeletons and their assistance
Source: Commun Eng. 2023 Jul 12;2:43. doi: 10.1038/s44172-023-00091-2 (PMC10956004; doi:10.1038/s44172-023-00091-2)
Supplement: Supplementary file 2 — Supplementary Information [file 44172_2023_91_MOESM2_ESM.pdf]

1 Supplementary Information

1.1 Supplementary Methods

We conducted a separate, smaller study in which two participants (Subjects 2 and 7) underwent the main study's protocol, but on level-ground instead of at a 10° incline. This was done to investigate how their MVs changed when the walking was made easier and more representative of exoskeleton use in the community. Both participants' MVs diminished when the task was level-ground walking—Subject 2's MV dropped from 36.35% to -28.10%, while Subject 7's MV dropped from 26.68% to 12.62% (Fig. 6). These results suggest that for everyday mobility tasks, which are likely to be more similar in difficulty to level-ground walking than uphill walking, energetic exoskeleton assistance may have less value to potential adopters. Furthermore, augmentative exoskeletons may thus provide maximal value during highly strenuous tasks. Future work should investigate if these results are consistent across more subjects and different walking conditions.

1.2 Supplementary Tables

Supplementary Table 1: Powered condition cumulative prices and marginal values.

Subject	MV (%)	Exo Powered ($)	Walking No Exo ($)		
			Day 1	Day 2	Day 3
1	1.1	13.3	13.5		
2	36.4	42.4	66.6		
3	26.1	15.5	20.9		
4	-13.9	17.7	15.1	15.9	
5	-8.0	25.0	23.2		
6	2.5	26.9	27.6		
7	49.3	36.5	72.0		
8	55.8	62.8	142.1		
9	11.7	11.4	12.9		
10	11.5	11.2	12.6		
11	14.9	17.4	20.6	19.8	20.7
12	-59.4	31.0	19.4		
13	-32.4	35.2	26.6		
14	18.0	11.5	14.0		
15	-46.6	33.4	22.0	23.3	23.1
16	26.1	14.9	19.2	20.2	20.9

Supplementary Table 2: Powered-off condition cumulative prices and marginal values.

Subject	MV (%)	Exo Powered Off ($)	Walking No Exo ($)
1	-0.5	13.60	13.50
2	-26.4	84.20	66.50
3	-3.5	21.30	20.90
4	-0.1	15.10	15.10
5	-7.4	24.90	23.20
6	17.4	22.80	27.60
8	-40.5	199.70	142.10
9	-26.6	16.30	12.90
12	-119.9	42.80	19.40
13	-112.0	56.30	26.60

Supplementary Table 3: Cumulative prices for participants who repeated the walking-no-exo condition

Subject	Average Cumulative Price ($)	St. Dev. Cumulative Price ($)	St. Dev. Cumulative Price (% of Average)	Prices ($)		
				Day 1	Day 2	Day 3
4	15.5	0.4	2.8	15.1	15.9	
11	20.4	0.4	2.0	20.6	19.8	20.7
15	22.8	0.6	2.6	22.0	23.3	23.1
16	20.1	0.7	3.3	19.2	20.2	20.9

1.3 Supplementary Figures

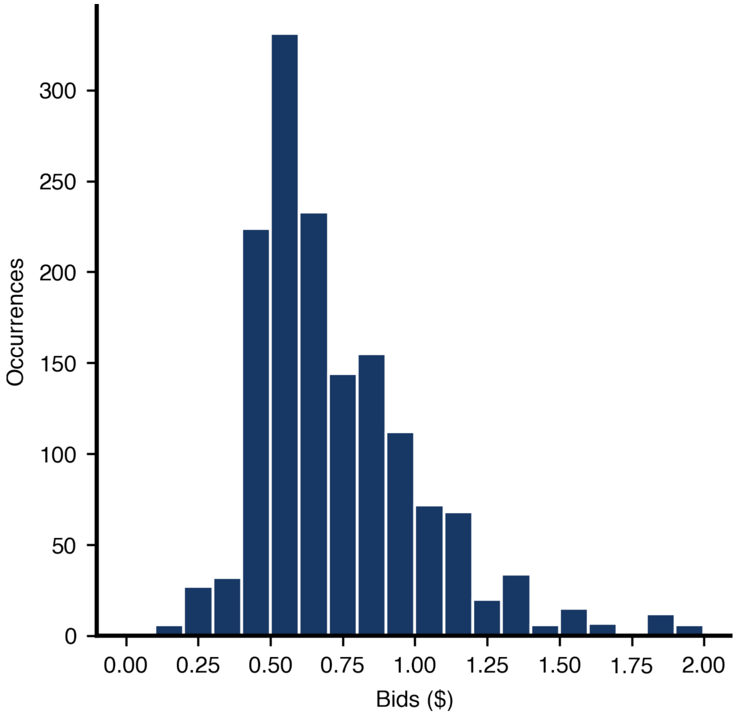

Supplementary Figure 1: A histogram of all the subject bids for the two-minute intervals across all tested conditions. The average bid was $0.75, with a standard deviation of $0.39.

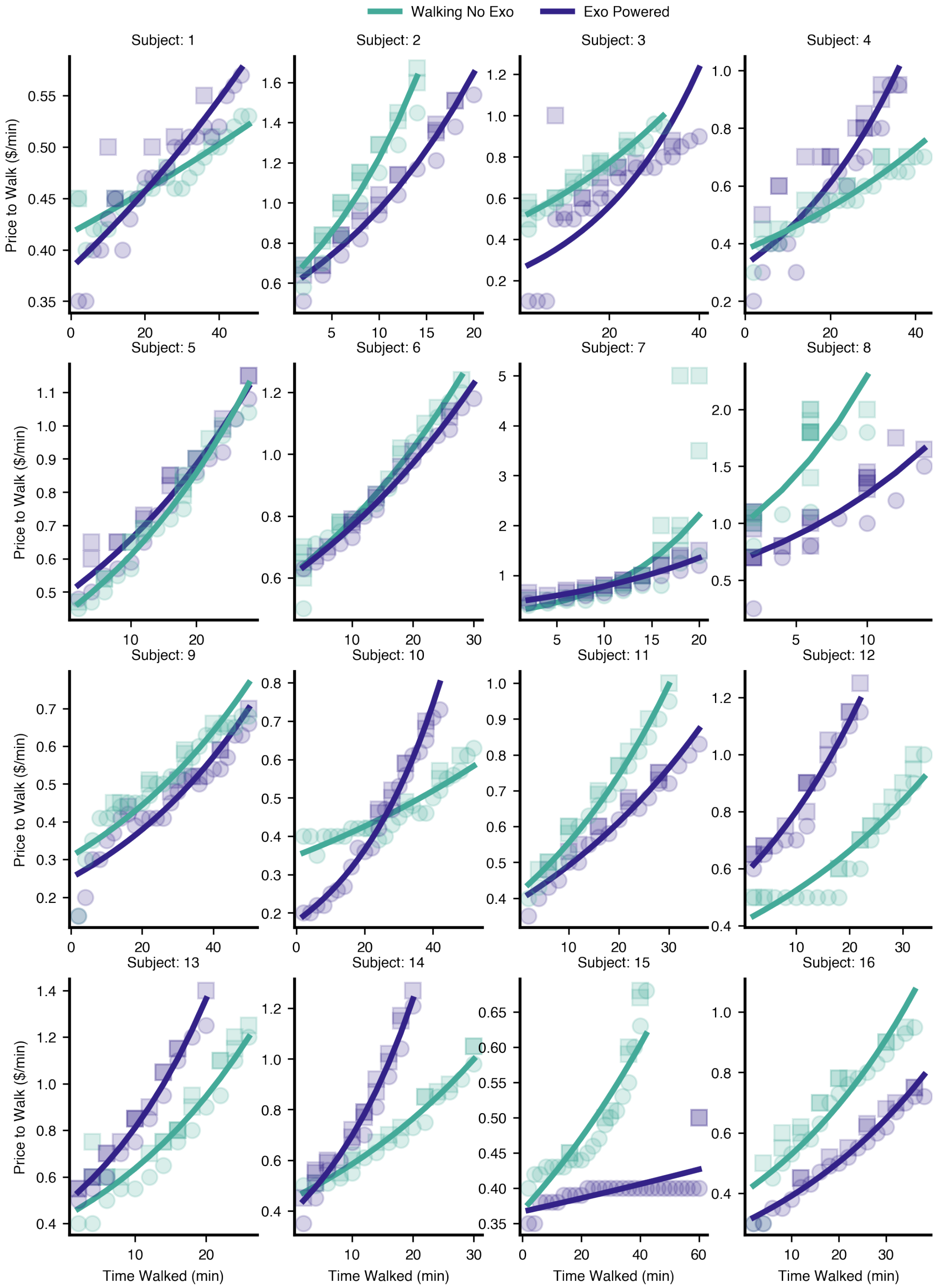

Supplementary Figure 2: The price to walk curves for the exo-powered (purple) and walking-no-exo (teal) from all sixteen subjects. Circles denote winning bids, while squares denote losing bids.

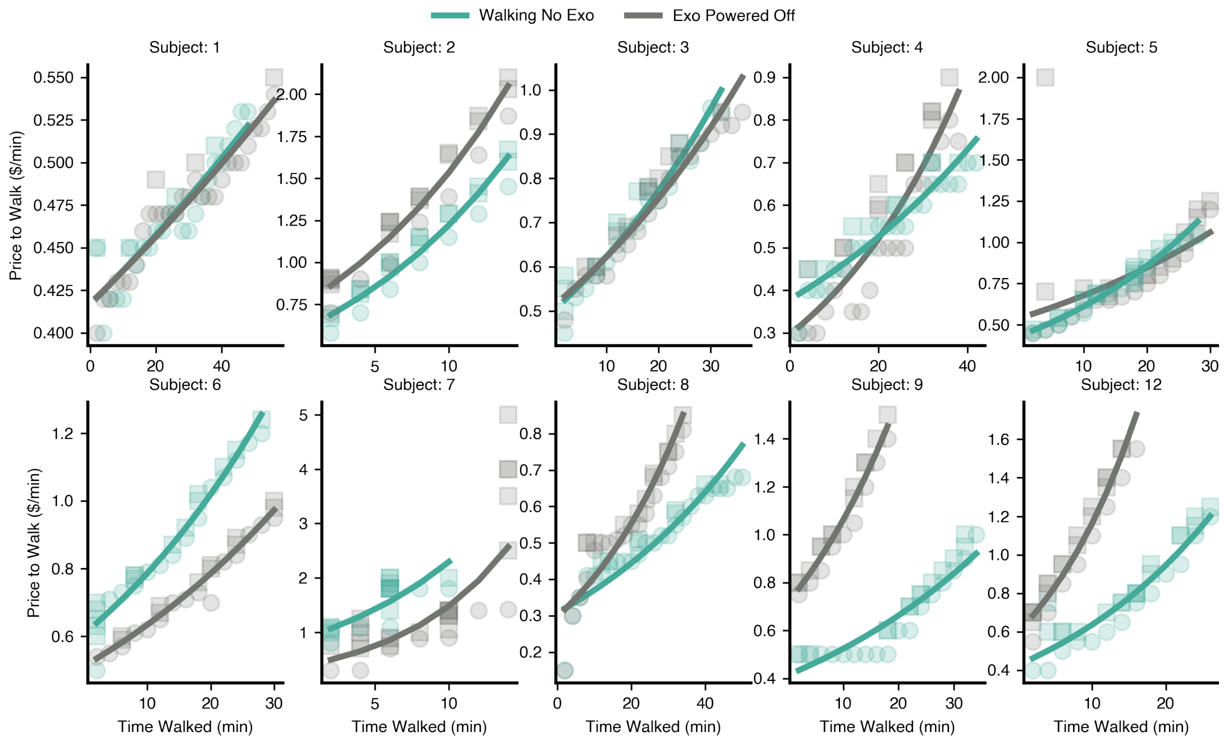

Supplementary Figure 3: The price to walk curves for the exo-powered-off (gray) and walking-no-exo (teal) for the ten subjects who completed the former condition. Circles denote winning bids, while squares denote losing bids.

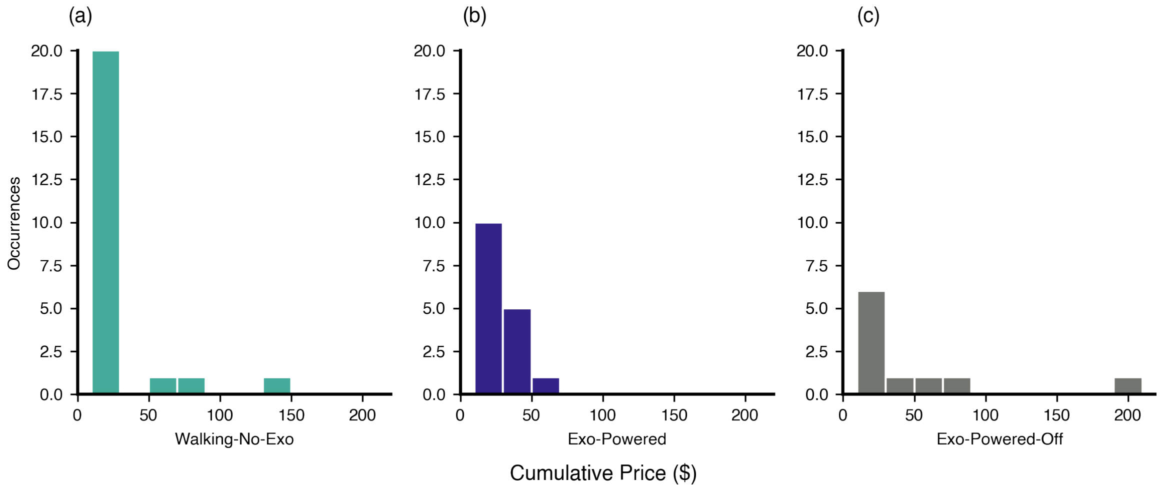

Supplementary Figure 4: Histograms of the cumulative costs during the different conditions. (a) The cumulative costs for the walking-no-exo condition. (b) The cumulative costs for the exo-powered condition. (c) The cumulative costs for the exo-powered off condition.

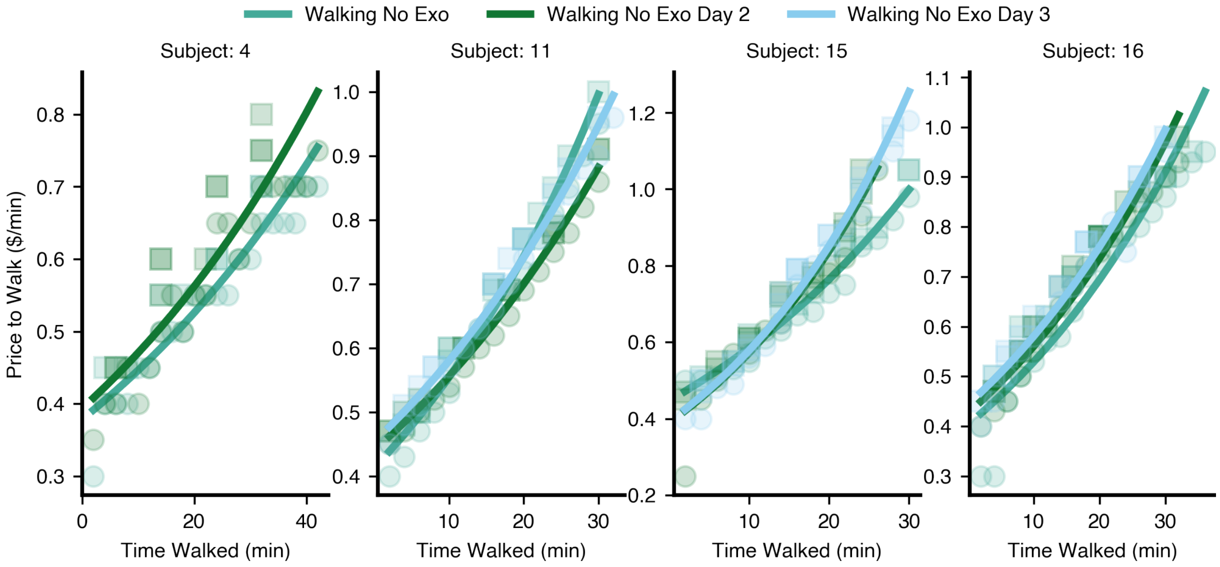

Supplementary Figure 5: The price to walk curves for the walking-no-exo conditions across different days for the four subjects who repeated the walking-no-exo condition. Circles denote winning bids, while squares denote losing bids. Subject 4 only repeated the walking-no-exo condition once, while the rest of the participants repeated it twice.

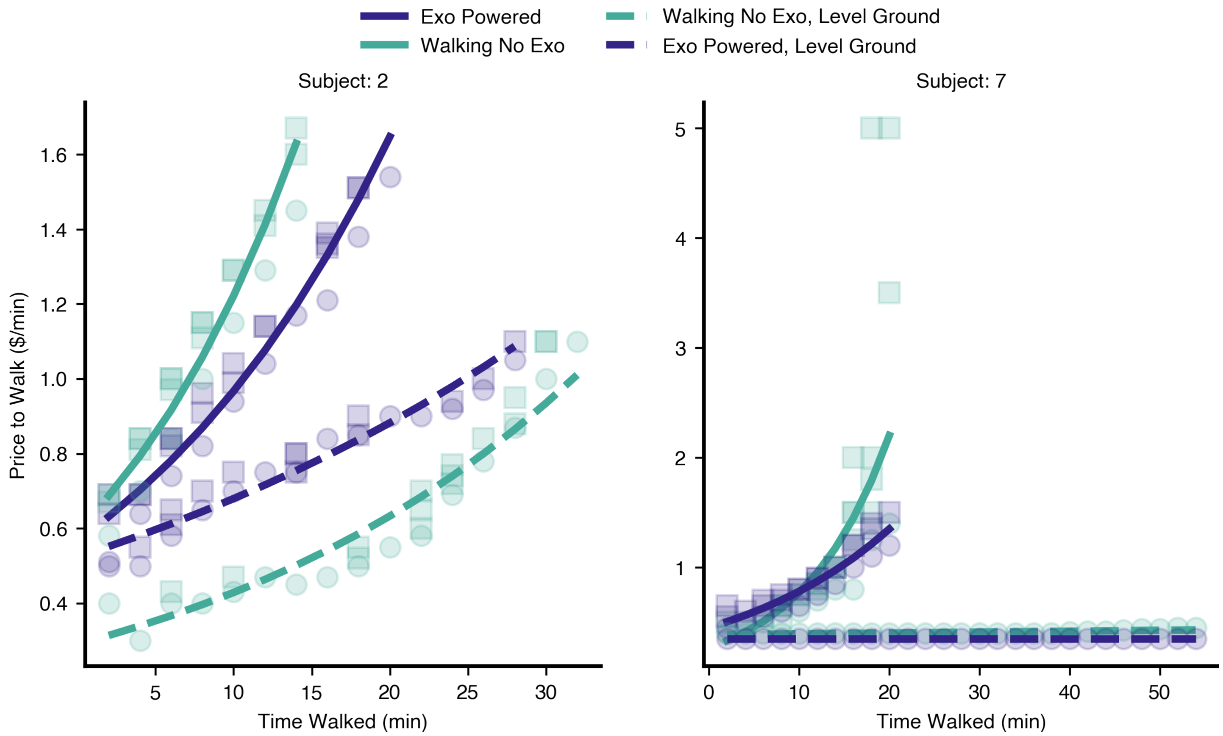

Supplementary Figure 6: The price to walk curves for two subjects (2 and 7) that repeated the walking-no-exo and exo-powered conditions on a level ground condition. The solid lines denote the standard 10° incline task featured in this work; the dashed lines are the level ground condition. Both participants' MVs decreased during the level ground condition.
