# Supplementary material for: The economic value of augmentative exoskeletons and their assistance
Source: Commun Eng. 2023 Jul 12;2:43. doi: 10.1038/s44172-023-00091-2 (PMC10956004; doi:10.1038/s44172-023-00091-2)
Supplement: Supplementary file 3 — Reporting Summary [file 44172_2023_91_MOESM3_ESM.pdf]

nature portfolio

Corresponding author(s): Elliott J. Rouse, Roberto Leo Medrano

Last updated by author(s): 5/11/2023

Reporting Summary

Nature Portfolio wishes to improve the reproducibility of the work that we publish. This form provides structure for consistency and transparency in reporting. For further information on Nature Portfolio policies, see our Editorial Policies and the Editorial Policy Checklist.

Statistics

For all statistical analyses, confirm that the following items are present in the figure legend, table legend, main text, or Methods section.

n/a	Confirmed	
☐	☒	The exact sample size (n) for each experimental group/condition, given as a discrete number and unit of measurement
☐	☒	A statement on whether measurements were taken from distinct samples or whether the same sample was measured repeatedly
☐	☒	The statistical test(s) used AND whether they are one- or two-sided<br>*Only common tests should be described solely by name; describe more complex techniques in the Methods section.*
☐	☒	A description of all covariates tested
☐	☒	A description of any assumptions or corrections, such as tests of normality and adjustment for multiple comparisons
☐	☒	A full description of the statistical parameters including central tendency (e.g. means) or other basic estimates (e.g. regression coefficient) AND variation (e.g. standard deviation) or associated estimates of uncertainty (e.g. confidence intervals)
☐	☒	For null hypothesis testing, the test statistic (e.g. F, t, r) with confidence intervals, effect sizes, degrees of freedom and P value noted<br>*Give P values as exact values whenever suitable.*
☒	☐	For Bayesian analysis, information on the choice of priors and Markov chain Monte Carlo settings
☒	☐	For hierarchical and complex designs, identification of the appropriate level for tests and full reporting of outcomes
☐	☒	Estimates of effect sizes (e.g. Cohen's d, Pearson's r), indicating how they were calculated

Our web collection on statistics for biologists contains articles on many of the points above.

Software and code

Policy information about availability of computer code

Data collection	Data was collected and stored using Python 3.7. and Microsoft Excel The programs used throughout this study are available via CodeOcean repository (link provided in paper).
Data analysis	Data was processed using Python 3.7. The programs used throughout this study are available via CodeOcean repository (link provided in paper).

For manuscripts utilizing custom algorithms or software that are central to the research but not yet described in published literature, software must be made available to editors and reviewers. We strongly encourage code deposition in a community repository (e.g. GitHub). See the Nature Portfolio guidelines for submitting code & software for further information.

Data

Policy information about availability of data

All manuscripts must include a data availability statement. This statement should provide the following information, where applicable:

- Accession codes, unique identifiers, or web links for publicly available datasets
- A description of any restrictions on data availability
- For clinical datasets or third party data, please ensure that the statement adheres to our policy

All data needed to evaluate the conclusions of the paper are available in the paper or the Supplementary Materials.

Human research participants

Policy information about studies involving human research participants and Sex and Gender in Research.

Reporting on sex and gender	Study results apply to all genders. Participants supplied their gender identities.
Population characteristics	Participants exhibited a wide range of ages, heights, weights, and activity levels.
Recruitment	Participants were recruited via word-of-mouth from around the University of Michigan Ann Arbor campus. Participant sampe may have skewed younger as a result.
Ethics oversight	All experiments were carried out with informed consent at the University of Michigan, with approval from the Institutional Review Board of the University of Michigan Medical School (IRBMED).

Note that full information on the approval of the study protocol must also be provided in the manuscript.

Field-specific reporting

Please select the one below that is the best fit for your research. If you are not sure, read the appropriate sections before making your selection.

☐ Life sciences ☒ Behavioural & social sciences ☐ Ecological, evolutionary & environmental sciences

For a reference copy of the document with all sections, see nature.com/documents/nr-reporting-summary-flat.pdf

Behavioural & social sciences study design

All studies must disclose on these points even when the disclosure is negative.

Study description	Study was quantitative experimental, and aimed to quantify the economic value of exoskeleton assistance during locomotion.
Research sample	Sample taken from the population of Ann Arbor and the University of Michigan campus. Sample is representative. Age, height, and weight information is included in the manuscript.
Sampling strategy	Sampling was conducted using convenience sampling methods. Sample size was chosen to exceed the typical sample size in an experiment that analyzes the effects of exoskeletons.
Data collection	Participants reported their data verbally, which was recorded via Microsoft Excel.
Timing	Data collection commenced March 2021 and concluded July 2021
Data exclusions	No data were excluded.
Non-participation	No participants dropped out
Randomization	All participants completed at least two trials: walking normally, and walking with exoskeleton assistance. Some participants were then randomly selected to either repeat the normal walking condition multiple times, or walk with the exoskeleton donned but not applying power.

Reporting for specific materials, systems and methods

We require information from authors about some types of materials, experimental systems and methods used in many studies. Here, indicate whether each material, system or method listed is relevant to your study. If you are not sure if a list item applies to your research, read the appropriate section before selecting a response.

Materials & experimental systems

n/a	Involved in the study
☒	☐ Antibodies
☒	☐ Eukaryotic cell lines
☒	☐ Palaeontology and archaeology
☒	☐ Animals and other organisms
☒	☐ Clinical data
☒	☐ Dual use research of concern

Methods

n/a	Involved in the study
☒	☐ ChIP-seq
☒	☐ Flow cytometry
☒	☐ MRI-based neuroimaging
